# Mechanisms of the Bushen Huoxue formula in the treatment of osteoarthritis based on network pharmacology-molecular targets

**DOI:** 10.1097/MD.0000000000029345

**Published:** 2022-08-12

**Authors:** Tingting Pang, Chang Liu, Junjie Yao, Jiahui Li, Zhongxu Li, Huijuan Lou, Siyuan Lei, Jiangchun Zhang, Li Dong, Yufeng Wang

**Affiliations:** a Department of Acupuncture and Tuina, Changchun University of Chinese Medicine, Changchun, China; b Department of Tuina, the Affiliated Hospital to Changchun University of Chinese Medicine, Changchun, China; c Department of Rehabilitation Medicine College, Changchun University of Chinese Medicine, Changchun, China.

**Keywords:** mechanisms, network pharmacology-molecular targets, osteoarthritis, the Bushen Huoxue formula

## Abstract

**Background:**

Osteoarthritis is a common degenerative disease with a high incidence, high disability rate, and poor prognosis. Clinical studies have shown that Bushen Huoxue formula can relieve joint swelling and pain and improve limb function and joint mobility, but there is a lack of high-quality scientific basis. Using network pharmacology and molecular docking technology to study the mechanism of Bushen Huoxue formula in the treatment of osteoarthritis.

**Methods:**

First, the active ingredients and corresponding target predictions of the formula were obtained through the Traditional Chinese Medicine Systems Pharmacology Database and Analysis Platform and the China National Knowledge Infrastructure. Meanwhile, the osteoarthritis disease targets were obtained through the genome annotation database platform (GeneCards) and the DrugBank database, and the target proteins obtained above were standardized using the Uniprot (https://www.uniprot.org) database standardization of names. Then, the Venn diagram was created by taking the intersection of the active ingredient and the target of the disease, and the “active ingredient-target” network was constructed and analyzed using Cytoscape 3.7.2 software. At the same time, the intersecting targets were imported into the Search Tool for the Retrieval of Interaction Gene/Proteins database to build a protein-protein interaction network and to screen the core targets; the intersecting targets were visualized by using the Database for Annotation, Visualization and Integrated Discovery 6.8 database for gene ontology functional analysis and the Kyoto Encyclopedia of Genes and Genomes (KEGG) pathway enrichment analysis, and construct the “active ingredient-target-pathway” network. Finally, the main active ingredients of the formula for tonifying the kidney and invigorating the blood were validated by molecular docking with the core targets.

**Results:**

A total of 194 active ingredients and 365 targets of the Bushen Huoxue formula were collected, 776 targets for osteoarthritis diseases and 96 targets for the intersection of active ingredients and diseases. The Kyoto Encyclopedia of Genes and Genomes enrichment analysis yielded 104 relevant pathways, including tumor necrosis factor signaling pathways, cancer signaling pathways, nucleotide-binding oligomerization domain-like receptor signaling pathways, Toll-like receptors signaling pathways, and osteoclast differentiation, apoptosis, T-cell receptor signaling pathway, and other related pathways. The molecular docking results showed good binding of the main active ingredients to the core targets.

**Conclusion:**

This study shows that the treatment of osteoarthritis involves multicomponent, multitarget, and multipathway processes. The mechanism of anti-inflammatory, antioxidant, inhibition of cartilage matrix degradation, and reduction of subchondral bone destruction may be an important mechanism for the therapeutic effect.

## 1. Introduction

Osteoarthritis (OA) is a common degenerative bone disease characterized by synovial lesions, cartilage degeneration, subchondral bone collapse, periarticular osteophytes, muscle atrophy and weakness, and progressive joint pain, swelling, restricted movement, and even deformity.^[1]^ Epidemiological studies have found^[2–4]^ that more than 50% of people over 65 years of age suffer from OA to varying degrees, with 8%–10% of these patients becoming disabled due to the disease, and the annual cost is increasing significantly, which places a heavy burden on families and society. Modern medical treatment of the disease mainly includes nonsteroidal anti-inflammatory drugs, glucocorticoids, analgesics, and joint injections, which only improve the symptoms but do not slow down the progression of the disease.^[5,6]^ The disease belongs to the category of “paralysis” and “bone paralysis” in Chinese medicine.^[7]^ According to Chinese medical theory, the pathogenesis of the disease is based on the deficiency of the Bushen Huoxue formula in the ligaments.^[8]^

Bushen Huoxue formula was first seen in Dacheng of traumatology. It is composed of 10 g of Radix rehmanniae, Radix Bupleurum, Semen Cuscutae, 3 g of Cortex Eucommiae, Fructus Lycii, Radix Angelicae sinensis, Cornu cervi pantotrichum, Herba Cistanches, Myrrh and Rhizoma dulcis, and 2 g of Safflower. The whole formula is effective in tonifying the kidneys and strengthening the bones, activating blood circulation, resolving blood stasis, and relieving swelling and pain. It has become a classic traditional Chinese medicine prescription for the treatment of OA. The formula is effective in relieving clinical symptoms such as swelling and pain in the joints, improving physical function and joint mobility, and improving the quality of life of patients in the middle and late stages.^[9,10]^ Modern pharmacological studies have found that tonifying the kidney and invigorating the blood improve periarticular microcirculation,^[11]^ reduce the release of inflammatory factors,^[12,13]^ scavenge oxygen-free radicals,^[14]^ inhibit the apoptosis of chondrocytes,^[15]^ and promote the repair of synovial membrane and chondrocytes for the treatment of OA.^[16,17]^ Although clinical and basic studies have confirmed that tonifying the kidney and invigorating the blood can effectively improve the symptoms of OA, it is difficult to systematically analyze the relationship between components, targets, and pathways in purely experimental studies, whereas network pharmacology is based on bioinformatics and studies the relationship between “multicomponent-multitarget-multipathway,” which is in line with the complexity of the Chinese medicine compound.^[18–21]^ Therefore, this article investigates the mechanism of action of the Bushen Huoxue formula in the treatment of OA by using network pharmacology and molecular docking techniques, and provides a scientific basis for revealing the effects of this Chinese medicine formula in the treatment of OA.

## 2. Methodology

### 2.1. Acquisition of active ingredients and corresponding targets of the formula for tonifying the kidney and invigorating the blood

The Traditional Chinese Medicine Systems Pharmacology Database and Analysis Platform (TCMSP) platform (https://tcmspw.com/tcmsp.php) was used to search the active ingredients of the formula for tonifying the kidney and invigorating the blood: Radix Rehmanniae, Semen Cuscutae, Cortex Eucommiae, Fructus Lycii, Radix Angelicae Sinensis, Cornu Cervi Pantotrichum, Herba Cistanches, Myrrh, Radix Dulcis, and Rhizoma Hong Hua, and according to the absorption, distribution, metabolism, excretion module, the active ingredients with oral bioavailability oral bioavailability ≥ 30% and drug-like index drug-like ≥ 0.18 were screened. The molecule identifications were entered into the Related Targets to find the corresponding biological targets. Among the active ingredients of bone marrow, the literature was reviewed to screen for those associated with OA. The names of the active ingredient targets were corrected with standard names using the Uniprot database (https://www.uniprot.org). The targets corresponding to the active ingredients of each drug were deweighted and imported into the Cytoscape 3.7.2 software to construct a “drug-active-ingredient-target” network.

### 2.2. Access to osteoarthritis-related targets

Using the GeneCards database (https://genealacart. genecards.org/) and the DrugBank database (https://go.drugbank.com/), the results were combined and deduplicated using “Osteoarthritis” as the search. The results of the 2 databases were combined and deduplicated using the GeneCards database and the DrugBank database, using “Osteoarthritis” as the search term. The names of the disease targets were corrected to standard names using the Uniprot database.

### 2.3. Production of “active ingredient-target” diagrams

The targets corresponding to the active ingredients of the formula were intersected with the targets corresponding to OA and visualized by VENNY 2.1 (https://bioinfogp.cnb.csic.es). The intersecting targets were imported into Cytoscape 3.7.2 software, and the “active ingredient-target” network diagram was drawn.

### 2.4. Construction and analysis of a network for the treatment of osteoarthritis with the formula of tonifying the kidney and activating blood

The 96 intersecting targets were imported into the Search Tool for the Retrieval of Interaction Gene/Proteins 11.0 database to obtain protein interactions in tab-separated values format, which were imported into Cytoscape 3.7.2 software to produce protein-protein interaction (PPI) network maps.

### 2.5. GO analysis and KEGG pathway analysis

The 96 intersecting targets between the Bushen Huoxue formula and OA were imported into the Database for Annotation, Visualization and Integrated Discovery database (http://david.ncifcrf.gov/), and gene ontology (GO) analysis and Kyoto Encyclopedia of Genes and Genomes (KEGG) pathway enrichment analysis were carried out, and the results were presented as bar graphs and bubble plots, respectively. In addition, the results were imported into the Cytoscape 3.7.2 software to obtain the “active ingredient-key target-signaling pathway” map.

### 2.6. Molecular docking

The 8 active ingredients with the highest degree of “active ingredient-intersection target” were molecularly docked to the 4 core targets with the highest degree of PPI network. The compounds required for docking were downloaded and checked in PubChem compound identification record and processed through Chembio 3D for Minimize Energy. The required 4 core targets and corresponding protein data banks (TNF-6OP0, interleukin [IL] 6-4O9H, v-akt murine thymoma viral oncogene homolog 1 [AKT1]-6S9X, mitogen-activated protein kinase 8 [MAPK8]-4IZY) were obtained from the Research Collaboratory for Structural Bioinformatics database (http://www.rcsb.org/), imported from Schrödinger software. Ligprep was used for compound ionization, desalting, generation of tautomers, setting of stereoisomers, and other pretreatments. Then docking with the optimized protein receptor was performed using the glidedock module. The results are expressed in terms of the binding energy generated by docking the compound to the protein. In general, the lower the binding energy of the compound to the protein receptor, the stronger its affinity.

## 3. Results

### 3.1. Screening of potential active ingredients and collection of corresponding targets

The TCMSP database was used to collect the single active ingredients of the formula for tonifying the kidney and invigorating the blood, with absorption, distribution, metabolism, excretion set to oral bioavailability ≥ 30% and drug-like ≥ 0.18. One hundred ninety-four active ingredients of the formula for tonifying the kidney and invigorating the blood were obtained, including 6 kinds of Cistanches, 2 kinds of Angelicae, 9 kinds of Douhu, 28 kinds of Eucommia, 45 kinds of Lycium, 22 kinds of Safflower, 45 kinds of Myrrh, 20 kinds of Cornus, 2 kinds of Radix Rehmanniae, 11 kinds of Cuscuta, and after the knowledge of the literature, After screening, 4 active ingredients of Boneset were identified. The molecule identifications of the active ingredients were entered into the Related Targets section of the TCMSP database, and the collected targets were corrected using the Uniprot database to remove duplicate genes, resulting in a total of 365 targets for the active ingredients of the Bushen Huoxue formula. The active ingredients and corresponding targets of the formula were imported into Cytoscape 3.7.2 to construct a “Chinese herbal medicine-active ingredient-target” map, and the nodes were sorted according to the degree value (Table 1). The proteins closely related to Bushen Huoxue formula include PTGS2, NCOA2, PGR, PTGS1, sodium voltage-gated channel alpha subunit 5, Nuclear receptor subfamily 3 group C member 2, gamma-aminobutyric acid type A receptor subunit alpha1, adrenoceptor beta 2, cholinergic receptor muscarinic 1, etc., which fully proved the close relationship between active components and targets and the relationship between multiple components and multiple targets of Bushi Decoction in vivo.

**Table 1:** Targets of action of active ingredients in the Bushen-Huoxue formula (degree ≥ 15).

Serial number	Gene name	Degree
1	*PTGS2*	87
2	*NCOA2*	79
3	*PGR*	65
4	*PTGS1*	54
5	*SCN5A*	39
6	*NR3C2*	39
7	*GABRA1*	38
8	*ADRB2*	35
9	*CHRM1*	31
10	*ADRA1B*	29
11	*KCNH2*	27
12	*CHRM2*	27
13	*RXRA*	27
14	*CHRM3*	26
15	*AR*	24
16	*CASP3*	23
17	*PRSS1*	23
18	*ADRA1A*	21
19	*BCL2*	21
20	*JUN*	21
21	*ACHE*	21
22	*F7*	19
23	*BAX*	19
24	*NCOA1*	19
25	*CASP9*	18
26	*ESR1*	18
27	*PDE3A*	17
28	*SLC6A4*	17
29	*OPRM1*	17
30	*CASP8*	17
31	*CHRM4*	16
32	*SLC6A2*	16
33	*PRKCA*	15
34	*PON1*	15
35	*NOS2*	15
36	*MAOB*	15

### 3.2. Disease target screening and Venn diagramming of intersecting targets

**Figure 1.**
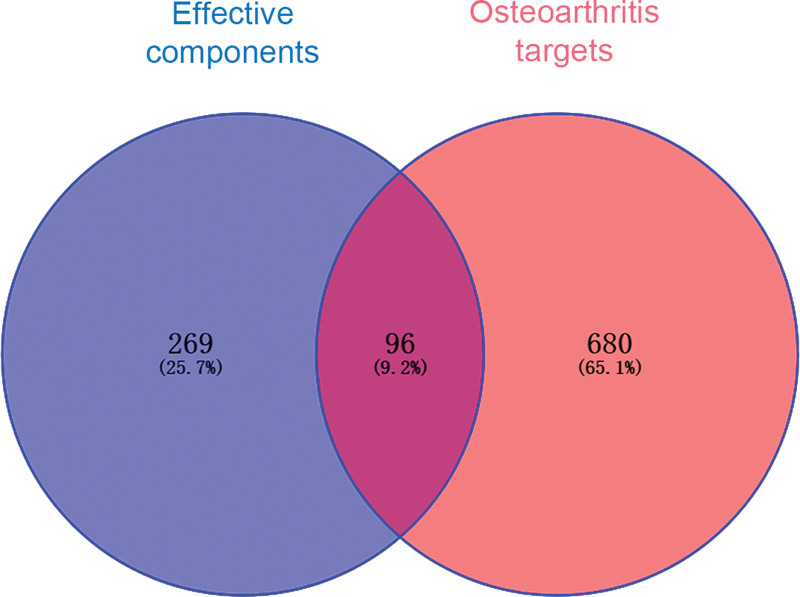
Venn diagram of the corresponding targets of the effective components of Bushen Huoxue formula and osteoarthritis targets.

A total of 776 OA targets were found using the GeneCard and DrugBank databases. The 194 active ingredients of the Bushen Huoxue formula corresponded to 365 targets, and the above targets were standardized by gene names using the Uniprot database. The above targets were normalized by using the Uniprot database. Using VENNY 2.1, 96 intersecting targets were obtained between the active ingredients of the Bushen Huoxue formula and OA, and the Venn diagram is shown in Figure 1.

### 3.3. “Active ingredient-target” network analysis

**Figure 2.**
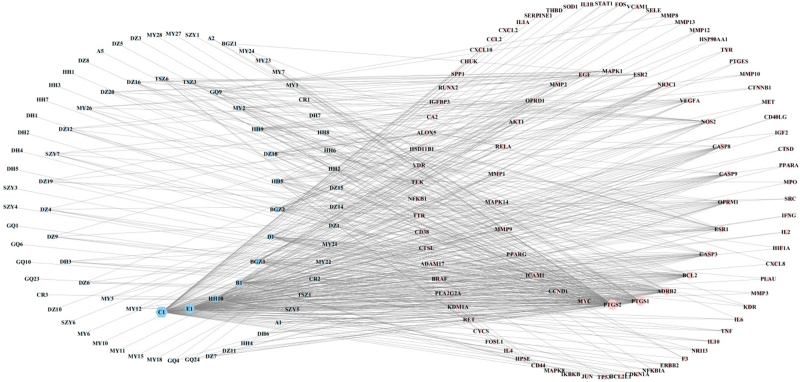
The network of “active ingredient—target.” MY represents Myrrh, HH represents Safflower, and so on. The hexagonal blue nodes represent active ingredients, the circular pink nodes represent intersection targets, and the gray lines represent edges.

The “active ingredient-target” network of 96 intersecting targets was plotted using Cytoscape 3.7.2 (Fig. 2). Network Analyzer analysis yielded 176 nodes (80 from compounds and 96 from gene targets) with 517 edges. The active ingredients with degree >15 were screened, as shown in Table 2.

**Table 2:** The top 8 main chemical components with degree value greater than 15.

Name in the text	Compound name in English	Degree value	Affiliated herbs
C1	Quercetin	89	Eucommiae Cortex, Carthami Flos, Carthami Flos, Cistanches Herba, Myrrha, Cuscutae Semen
E1	Beta-sitosterol	64	Eucommiae Cortex, Carthami Flos, Angelicae Pubescentis Radix, Corni Fructus, Angelicae Sinensis Radix, Carthami Flos, Cistanches Herba, Myrrha, Cuscutae Semen
HH10	Luteolin	31	Carthami Flos
B1	Kaempferol	29	Eucommiae Cortex, Carthami Flos, Cuscutae Semen
BGZ3	Isobavachin	24	Psoraleae Fructus
D1	Stigmasterol	23	Carthami Flos, Corni Fructus, Angelicae Sinensis Radix, Lycii Fructus, Rehmanniae Radix Praeparata, Myrrha, Psoraleae Fructus
BGZ2	Coumestrol	21	Psoraleae Fructus
HH5	Baicalein	17	Carthami Flos

### 3.4. Construction and analysis of PPI networks

**Figure 3.**
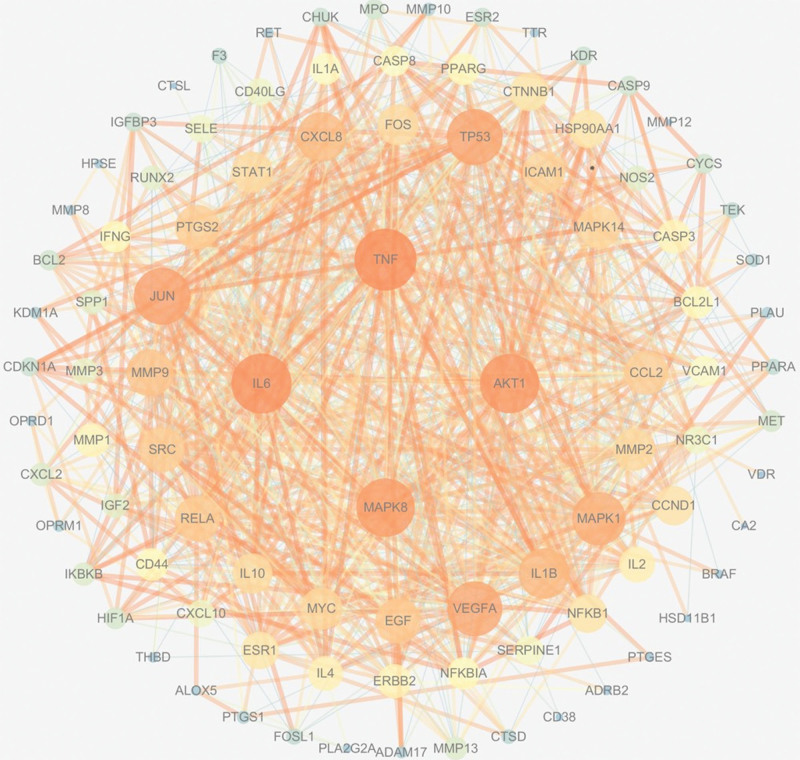
The interaction network of potential targets of Bushen Huoxue formula in the treatment of osteoarthritis. Genes are represented by circles; the larger the degree, the larger the shape and the darker the color; the larger the number of related links between genes, the darker the line. AKT1 = v-akt murine thymoma viral oncogene homolog 1, IL6 = interleukin-6, JUN = jun proto-oncogene, AP-1 transcription factor subunit, MAPK8 = mitogen-activated protein kinase 8, TNF = tumor necrosis factor, TP53 = tumor protein P53, VEGFA = vascular endothelial growth factor A.

The 96 intersecting targets were imported into the Search Tool for the Retrieval of Interaction Gene/Proteins database and screened for genes meeting a confidence level >0.7, showing a total of 96 targets with 866 edge counts in the PPI. Ninety-six targets were visualized in the PPI network graph using Cytoscape 3.7.2 (Fig. 3). The targets with degree >30 were screened, as shown in Table 3.

**Table 3:** The targets of Bushen-Huoxue formula in the treatment of osteoarthritis (degree > 30).

Serial number	Gene name	Degree
1	*TNF*	54
2	*IL6*	52
3	*AKT1*	51
4	*MAPK8*	50
5	*JUN*	48
6	*TP53*	47
7	*VEGFA*	46
8	*MAPK1*	44
9	*IL1B*	41
10	*CXCL8*	40
11	*MMP9*	37
12	*EGF*	35
13	*SRC*	35
14	*RELA*	35
15	*CCL2*	34
16	*PTGS2*	34
17	*MAPK14*	32
18	*ICAM1*	32
19	*MMP2*	31
20	*MYC*	31
21	*FOS*	30
22	*STAT1*	30

### 3.5. GO analysis and KEGG pathway analysis

**Figure 4.**
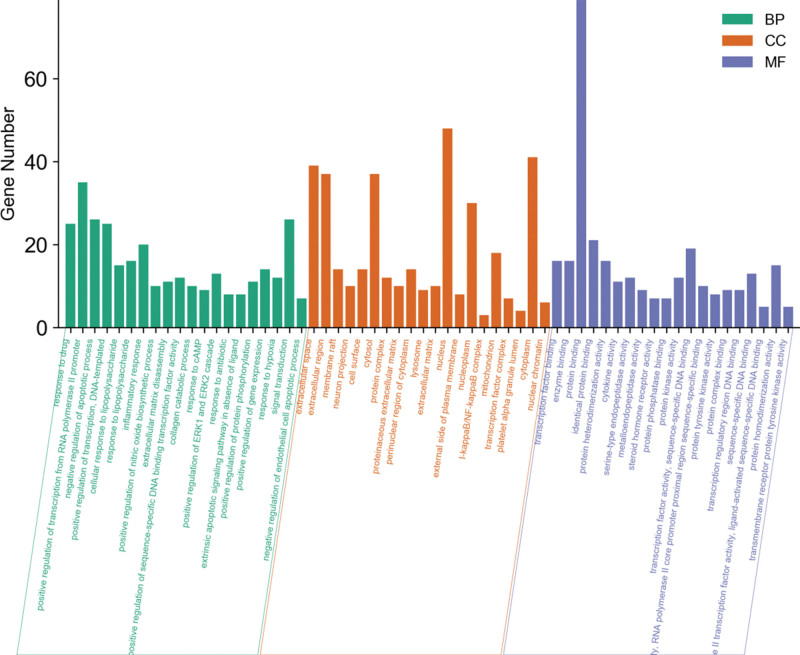
Go enrichment analysis (top 20 of *P* value). BP = biological process, CC = cell composition, DNA = deoxyribonucleic acid, ERK = extracellular-signal-regulated kinases, MF = molecular function, RNA = ribonucleic acid.

**Figure 5.**
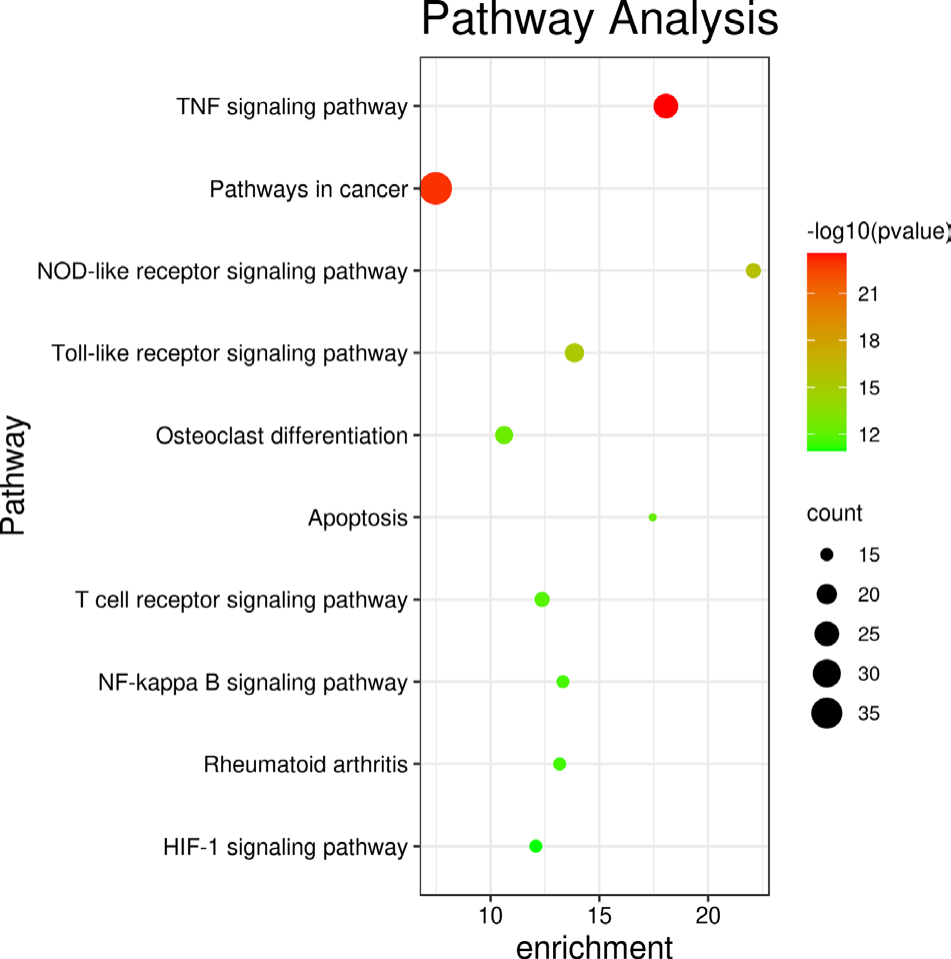
KEGG pathway analysis (top 10 *P* values). HIF = hypoxia-inducible factor, KEGG = kyoto encyclopedia of genes and genomes, NF = nuclear factor, NOD = nucleotide-binding oligomerization domain, TNF = tumor necrosis factor.

**Figure 6.**
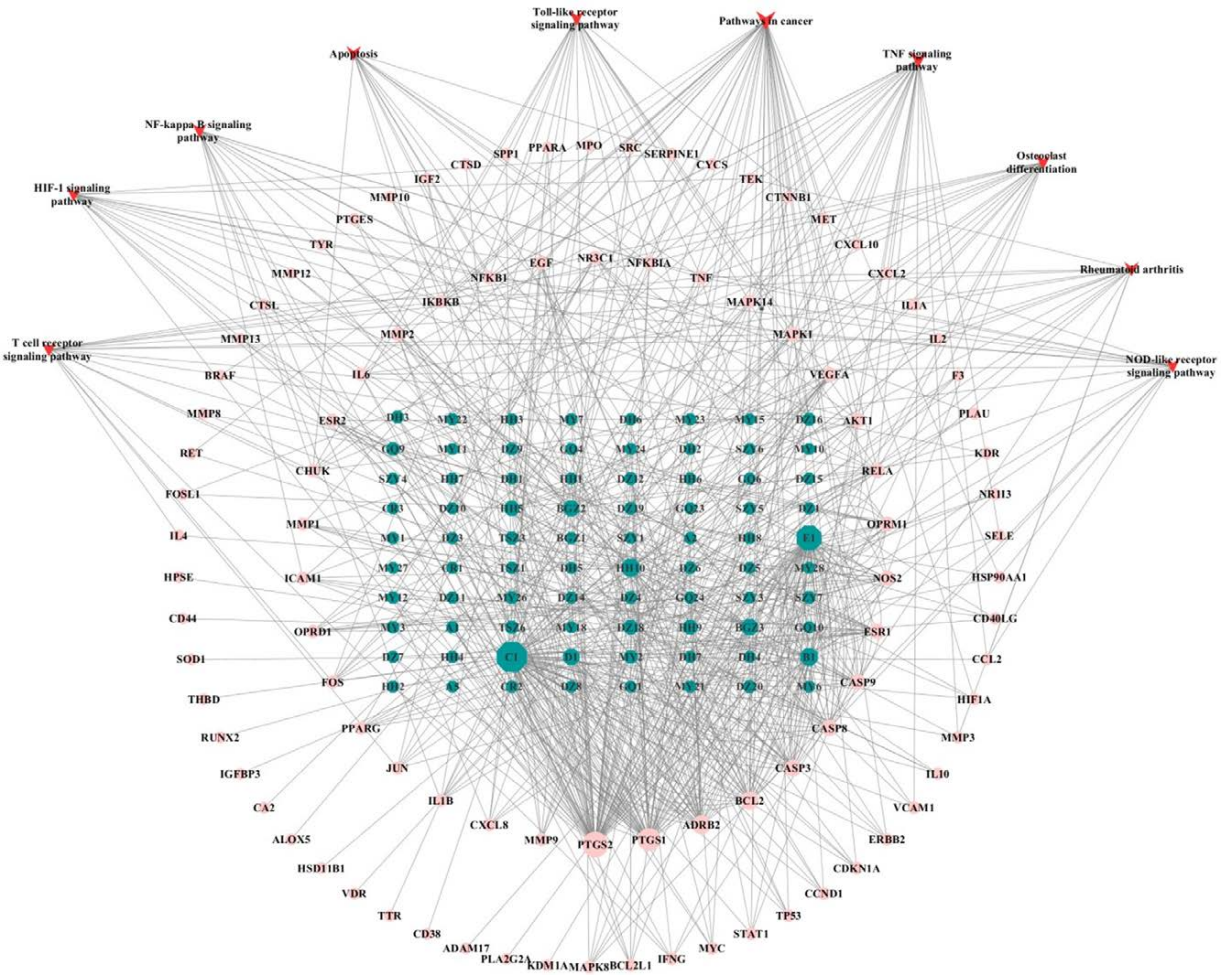
Network of active components-key target-signaling pathway. The hexagonal green nodes represent drug active ingredients, the circular pink nodes represent key targets, and the triangular red nodes represent pathways. HIF = hypoxia-inducible factor, NF = nuclear factor, NOD = nucleotide-binding oligomerization domain, TNF = tumor necrosis factor.

The intersecting targets the Bushen Huoxue formula and OA were imported into the Database for Annotation, Visualization and Integrated Discovery 6.8 database for GO analysis and KEGG pathway enrichment analysis. A total of 479 biological processes were obtained, mainly related to the response to drugs, positive regulation of ribonucleic acid polymerase II promoter transcription, negative regulation of apoptosis process, inflammatory response, positive regulation of nitric oxide biosynthesis process, and degradation of extracellular matrix, collagen. The cell composition obtained 48 pathways, mainly related to extracellular space and region, cytoplasmic lysis, nucleus, cytoplasm, cytoplasmic membrane, etc.; the molecular function obtained 80 pathways, mainly related to transcription factor binding, enzyme binding, protein binding, cytokine activity, and other functions (Fig. 4). One hundred four related pathways were obtained by KEGG enrichment analysis. The pathways related to OA were screened by reviewing the literature, including TNF signaling pathway, cancer signaling pathway, nucleotide-binding oligomerization domain-like receptor signaling pathway, Toll-like receptor (TLR) signaling pathway, osteoclast differentiation, apoptosis, and T cell receptor signaling pathway (Fig. 5). The Network of active components-key target-signaling pathway was plotted using the Cytoscape 3.7.2 software (Fig. 6). The treatment of OA with the Bushen Huoxue formula is based on 194 active ingredients acting on 10 major pathways and 96 target genes. This shows that the formula works through multiple components, multiple pathways, and multiple targets in the treatment of OA.

### 3.6. Molecular docking analysis

**Figure 7.**
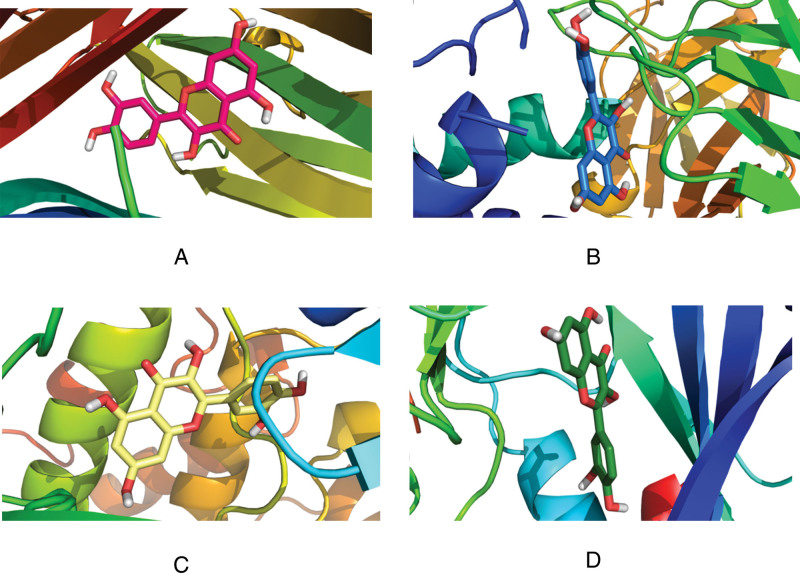
Three-dimensional diagram of quercetin docking with target molecules. (A) TNF. (B) IL6. (C) AKT3. (D) MAPK8. AKT3 = AKT serine/threonine kinase 3, IL6 = interleukin-6, MAPK8 = mitogen-activated protein kinase 8, TNF = tumor necrosis factor.

**Figure 8.**
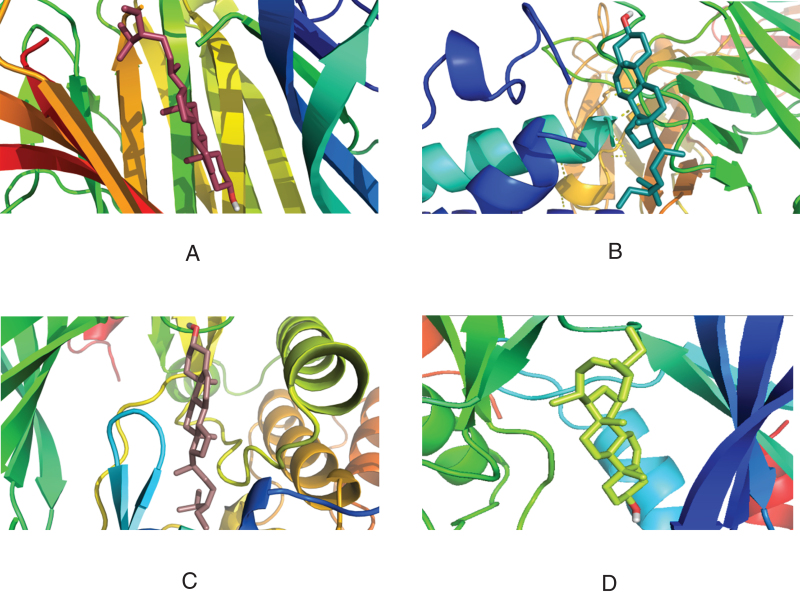
Three-dimensional docking of β-sitosterol with target molecules. (A)TNF. (B) IL6. (C) AKT3. (D) MAPK8. AKT3 = AKT serine/threonine kinase 3, IL6 = interleukin-6, MAPK8 = mitogen-activated protein kinase 8, TNF = tumor necrosis factor.

**Figure 9.**
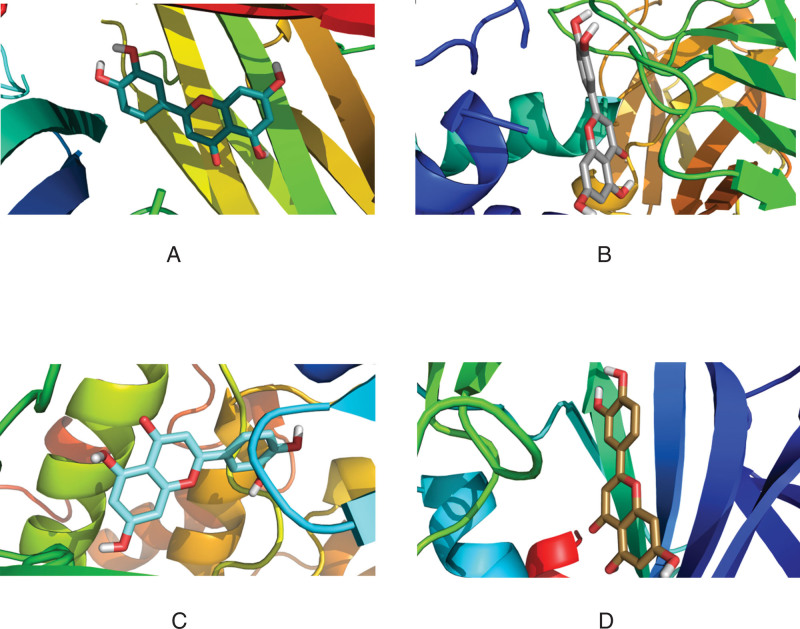
Three-dimensional docking of luteolin with target molecule. (A)TNF. (B) IL6. (C) AKT3. (D) MAPK8. AKT3 = AKT serine/threonine kinase 3, IL6 = interleukin-6, MAPK8 = mitogen-activated protein kinase 8, TNF = tumor necrosis factor.

**Figure 10.**
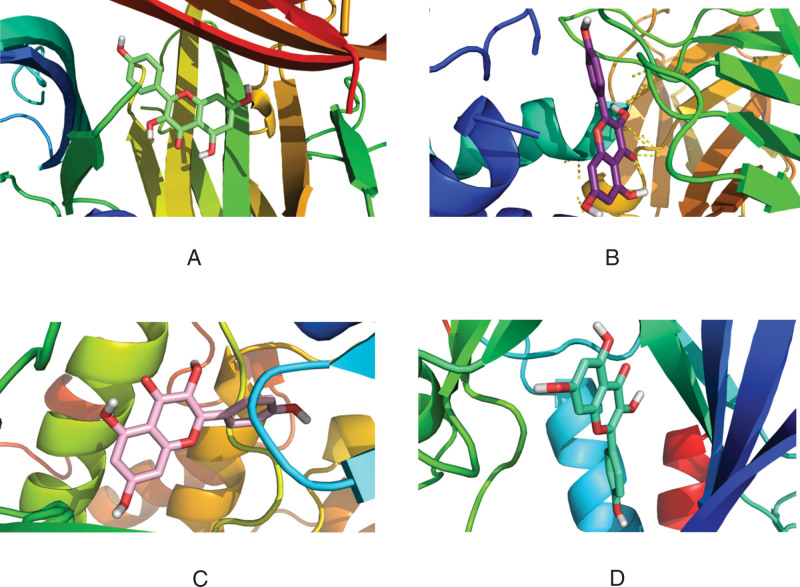
Three-dimensional view of kaempferol docking with target molecule. (A)TNF. (B) IL6. (C) AKT3. (D) MAPK8. AKT3 = AKT serine/threonine kinase 3, IL6 = interleukin-6, MAPK8 = mitogen-activated protein kinase 8, TNF = tumor necrosis factor.

The four core proteins (TNF, IL6, AKT1, mapk8) in the PPI network were docking with the eight active components (quercetin, beta sitosterol, luteolin, kaempferol) with the highest moderate value in the "active component intersection target" network. The molecular docking was performed with the 8 active ingredients (quercetin, beta-sitosterol, luteolin, and kaempferol) with the highest values in the “active ingredient-intersection target” network. The docking results are shown in Table 4. Except for the binding energy of −4.4 KJ·mol−^1^ for beta-sitosterol and IL-6, the binding energy of all the other active ingredients was less than −7.0 KJ·mol−^1^, which showed good binding power. The above results were imported into the Pymol software for visualization, and the 3D images were output (Figs. 7–10).

**Table 4:** Binding energy of active component and targets molecule docking protein.

Compound	CAS serial number	Binding energy/KJ·mol^−1^			
		*TNF(6O90*)	*IL6(4O9H*)	*AKT1(6S9X*)	*MAPK8(4IZY*)
Quercetin	117-39-5	−9.0	−7.7	−9.7	−7.5
Beta-sitosterol	83-46-5	−9.2	−4.4	−11.4	−8.0
Luteolin	491-70-3	−8.9	−7.3	−10.0	−7.5
Kaempferol	520-18-3	−8.8	−7.5	−9.4	−7.3

## 4. Discussion

In order to investigate the mechanism of action of this formula in the treatment of OA, this study used network pharmacology as the research method and molecular docking to validate the research results. A total of 194 active ingredients of the Bushen Huoxue formula were screened, and 96 key targets were obtained by intersecting the corresponding targets of active ingredients and OA targets. According to the “active ingredient-intersecting targets” network diagram, the highest active ingredients of Kidney and Blood Formula for OA treatment include quercetin, β-sitosterol, lignan, kaempferol, isoptin dihydroxyflavone, dousterol, coumarin, and baicalin. This reflects the multicomponent and multitarget characteristics of the Bushen Huoxue formula for OA treatment. Quercetin has anti-inflammatory and antioxidant effects. Animal studies have found that,^[22,23]^ after intraperitoneal injection of quercetin in osteoarthritic rats, it can activate the silent information regulator/AMP-activated protein kinase signaling pathway to inhibit endoplasmic reticulum stress-induced chondrocyte apoptosis. In addition, quercetin reduced serum *IL-1β* and *TNF-α* cytokine concentrations in OA mice, reducing the inflammatory response and maintaining the integrity of articular cartilage. β-sitosterol, dousterol, and coumarin are phytoestrogen-like substances that inhibit osteolysis of cartilage and subchondral bone by regulating nuclear factor kappa-B and osteoprotegerin/receptor activator of NF-κB/receptor activatorof NF-κB ligand signaling pathways, maintaining a dynamic balance between bone resorption and bone formation in the subchondral bone of the joint, thereby delaying the progression of arthritis.^[24–26]^ Lignans and isochondroitin dihydroflavonoids are natural flavonoids with strong anti-inflammatory effects. It was found that lignans can effectively inhibit OA chondrocyte proliferation, downregulate the expression of c-Jun N-terminal kinase and p38 mitogen-activated protein kinase in chondrocytes, downregulate the expression of nitric oxide, *TNF-α*, and IL-6, reduce the inflammatory response, protect chondrocytes, and slow down cartilage degeneration.^[27]^ It also inhibited the expression of nitric oxide synthase and cyclo-oxygenase-2, as well as inhibiting mitogen-activated protein kinase-related extracellular signal-regulated kinase and p38 signaling pathways to reduce inflammation in osteoarthritic chondrocytes.^[28,29]^ Baicalin can improve pain and expand a range of motion in OA model dogs, whereas targeted regulation of AKT1 reduces collagen degradation by decreasing the expression of proinflammatory factors *IL-1β* and *TNF-α* and decreasing the expression of cartilage matrix degradation-related enzymes matrix metalloproteinase-13 and a disintegrin and metalloproteinase with thrombospondin motif-5.^[30]^ The active ingredients of the Bushen Huoxue formula mainly include flavonoids and phytosterols, which delay chondrocyte apoptosis and reduce the destruction and resorption of articular cartilage and subchondral bone through anti-inflammatory and antioxidant pharmacological effects.

The targets screened for PPI intersection network core protein degree values greater than 50 were TNF, IL6, AKT1, and MAPK8. TNF and IL6 play a key role in the progression of inflammation. The current study found that *TNF-α* and IL-6 interact with each other and both promote the proliferation of synovial cells, which stimulates the production of matrix metalloproteinases and other cytotoxins and leads to bone erosion and cartilage destruction.^[31]^ Moulharat et al^[32]^ found that *TNF-α* increased the production of cyclooxygenase-2, IL-6, IL-8, monocyte chemoattractant protein-1, and regulated upon activation normal T cell expressed and secreted in chondrocytes, as well as prostaglandin E2 and nitric oxide levels, increased levels of matrix degrading proteases MMP-1, MMP-3, MMP-9, MMP-13, ADAMTS-4, and a disintegrin and metalloproteinase with thrombospondin motif-5, and inhibited synthesis of type II collagen and aggregates, leading to degradation of chondrocyte matrix components, collagen breakage, and joint destruction. In addition, both stimulate recruitment of articular leukocytes and receptor activator of NF-κB ligand differentiation, promote osteoblast maturation and differentiation, reduce chondrocyte proliferation, lead to disruption of bone homeostasis in articular cartilage and subchondral bone, and aggravate the progression of OA.^[33]^ The phosphoinositol-3 kinase-protein kinase B signaling pathway is one of the important pathways involved in apoptosis, and AKT1 is the most closely associated downstream factor with chondrocytes in this pathway. Current studies have found that AKT1 regulates neutrophils to induce their release of more inflammatory factors, and in addition, it inhibits pyrophosphate levels in chondrocytes to promote cartilage calcification.^[34]^

GO analysis results show that the key target gene-enrichment biological processes in the treatment of OA with the Bushen Huoxue formula are mainly related to inflammation, positive regulation of nitric oxide biosynthesis process, degradation of extracellular matrix, collagen catabolic process, etc., which are all related to inflammation, oxidative stress, destruction of articular cartilage, and subchondral bone. TLRs are a general term for a group of pattern recognition receptors, and it has been found that activation of these pathways can produce *TNF-α*, IL-1, and other inflammatory factors, causing local inflammatory damage to the joint. In addition, TLR4 in the pathway binds to damage factors released from injured tissues, activating nuclear factor kappa-B and promoting the release of inflammatory factors, further aggravating the degree of joint damage.^[35]^ The subchondral bone of the joint disperses the mechanical load on the joint, while providing nutrients to the adjacent cartilage through the terminal vessels and secreting cytokines to maintain cartilage metabolism.^[36]^ It is found that the activation of osteoclast differentiation leads to the secretion of various proteases and cytokines, resulting in bone resorption, bone destruction, loss of trabeculae, and fracture of the subchondral bone, disrupting the biomechanical balance of the subchondral bone, and promoting the release of inflammatory factors.^[37]^ The pathological changes in the synovial, cartilage, and subchondral bone are not described in this article.

The molecular binding energy between ligand and receptor was found to be less than −7 KJ mol−^1^, except for IL-6 and β-sitosterol, which means that the active ingredients of the Bushen Huoxue formula can bind to the core target protein stably, indicating that the Bushen Huoxue formula can exert its therapeutic effect on OA by inhibiting the release of inflammatory mediators, regulating chondrocyte apoptosis, and modulating immune activity.

Although our study explored the mechanism of Bushen Huoxue formula in the treatment of OA, there are still some limitations. First of all, the research data are obtained from the literature, so we can not determine the authenticity and integrity of the results. Secondly, the quality of literature is not high enough, and there are few risk assessment projects that can be clearly put forward. In addition, further experiments are needed to confirm the prediction results.

In this article, the mechanism of the formula for treating OA by tonifying the kidney and invigorating the blood was demonstrated through a network pharmacology approach and validated by molecular docking technology. It was demonstrated that the formula exerts its therapeutic effects on OA through multiple components, multiple targets and multiple pathways. The active ingredients in the formula, including quercetin, β-sitosterol, lignan, kaempferol, isopsoralen dihydroxyflavone, dousterol, coumarin, and baicalin, act on key target proteins such as TNF, IL6, AKT1, and MAPK8 to regulate the TNF signaling pathway, TLR signaling pathway, osteoclast differentiation, and other signaling pathways, thus acting as anti-inflammatory, antioxidant, inhibiting cartilage matrix degradation, and reducing subchondral bone destruction. It is a synergistic treatment for OA by inhibiting the degradation of cartilage matrix and reducing the destruction of subchondral bone.

## Acknowledgements

We thank Prof. Yufeng Wang for his critical guidance and problem solving for this thesis and all the people in this study.

## Author contribution

Main analysis and drafting of the article: Tingting Pang, Yufeng Wang, and Li Dong.

Research design: Chang Liu and Huijuan Lou.

Introduction and discussion sections: Jiahui Li, Siyuan Lei, and Jiangchun Zhang.

Article preparation: Junjie Yao and Zhongxu Li.

All authors wrote, read, and approved the article. Conceptualization: Yufeng Wang and Li Dong. Data curation: Tingting Pang, Chang Liu, and Huijuan Lou.

Formal analysis: Tingting Pang, Junjie Yao, Jiahui Li, and Siyuan Lei.

Funding acquisition: Tingting Pang and Chang Liu.

Investigation: Tingting Pang, Junjie Yao, Yufeng Wang, and Li Dong. Methodology: Tingting Pang, Yufeng Wang, and Chang Liu.

Project administration: Yufeng Wang and Li Dong.

Resources: Tingting Pang, Chang Liu, Junjie Yao, and Zhongxu Li. Software: Tingting Pang, Jiahui Li, and Jiangchun Zhang.

Supervision: Yufeng Wang and Li Dong.

Validation: Yufeng Wang, Li Dong, Junjie Yao, and Jiahui Li.

Visualization: Chang Liu.

Writing-original draft: Tingting Pang, Yufeng Wang, and Jiahui Li.

Writing-review and editing: Tingting Pang, Yufeng Wang, and Li Dong.
